# ﻿The genus *Climaciella* Enderlein, 1910 (Neuroptera, Mantispidae) in French Guiana

**DOI:** 10.3897/zookeys.1153.95960

**Published:** 2023-03-10

**Authors:** Adrian Ardila-Camacho, Shaun L. Winterton, Atilano Contreras-Ramos

**Affiliations:** 1 Posgrado en Ciencias Biológicas, sede Instituto de Biología, Universidad Nacional Autónoma de México (UNAM), Apdo. Postal 70-153, 04510 Ciudad de México, Mexico; 2 California State Collection of Arthropods, California Department of Food & Agriculture, Sacramento, 95832 CA, USA; 3 Colección Nacional de Insectos, Departamento de Zoología, Instituto de Biología, Universidad Nacional Autónoma de México (UNAM), Apdo. Postal 70-153, 04510 Ciudad de México, Mexico

**Keywords:** Lacewings, Neotropics, taxonomy

## Abstract

The genus *Climaciella* Enderlein, 1910 is a remarkable group of mantidflies (Neuroptera: Mantispidae: Mantispinae) distributed from Canada to Argentina, including parts of the Caribbean. This genus comprises nine valid extant species plus an extinct species from the late Oligocene of France. Species exhibit Batesian mimicry with vespid wasps (Vespidae). Herein, six species of *Climaciella* from French Guiana are documented. Before this study only *C.semihyalina* (Le Peletier de Saint Fargeau & Audinet-Serville in Latreille et al. 1825) was known from this territory. Two new species, *C.elektroptera* Ardila-Camacho, Winterton & Contreras-Ramos, **sp. nov.** and *C.nigriflava* Ardila-Camacho, Winterton & Contreras-Ramos, **sp. nov.**, are described as well as the first records of *C.amapaensis* Penny, 1982, and *C.tincta* (Navás, 1914) provided from French Guiana. An unknown species recorded from a single female specimen is also presented. Based on the examination of material of *C.amapaensis* recorded here, a specimen previously recorded from Colombia as belonging to this species is herein proposed as a new species, *C.risaraldensis* Ardila-Camacho, **sp. nov.** A taxonomic key and high-resolution images of the species from French Guiana are provided.

## ﻿Introduction

Mantispidae (mantidflies) are a family of predatory insects included within the superfamily Mantispoidea (Winterton et al. 2018). This group is distinguished by the anteriorly curved ocular plane, a well-developed laminatentorium, a tubular pronotum set with maculae, and with forelegs inserted on an expanded anterior apex, presence of precoxal bridge and narrow-shaped postfurcasterna (Ardila-Camacho et al. 2021). On the forefemur, two primary processes on distal half of posteroventral row of processes, and a major, basal process on the anteroventral row of processes are present (Ardila-Camacho et al. 2021). The traditional classification scheme of Mantispidae proposed by Lambkin (1986a, b) considered four subfamilies, namely Symphrasinae, Drepanicinae, Calomantispinae, and Mantispinae, of which the former was always interpreted as sister of the remainder. Nonetheless, recent molecular and morphological studies recovered Symphrasinae as a subfamily of Rhachiberothidae, making Mantispidae non-monophyletic (Winterton et al. 2018; Ardila-Camacho et al. 2021). Even though thorough morphological data (Ardila-Camacho et al. 2021) and genomic scale data (Winterton et al. 2018) support the new position of Symphrasinae, several authors who recently described extinct species of Mantispoidea (Nakamine et al. 2021; Jouault 2022; Jouault et al. 2022; Baranov et al. 2022; Li et al. 2022) still follow the classification of Lambkin (1986a, b). In addition, previous phylogenetic studies based on different data systems (Lambkin 1986a; Liu et al. 2015; Shi et al. 2019; Lu et al. 2020) recovered Drepanicinae as sister to Calomantispinae + Mantispinae; however, the same aforementioned studies support Mantispinae as sister to Drepanicinae + Calomantispinae, with the latter two probably representing a single subfamily (Winterton et al. 2018; Ardila-Camacho et al. 2021).

Among the raptorial Mantispoidea, Mantispinae is the most diverse and widely distributed subfamily (Ohl 2004). This is a highly derived group, whose monophyly is supported by characters of the foreleg such as a ring-shaped groove on the coxa, a semi-triangular femur with anteroventral row of processes reduced to the major process, the anterior surface of the tibia is covered with clavate setae nearly over the entire extension, and the pretarsus is reduced to a single, simple claw (Ardila-Camacho et al. 2021). Currently, the subfamily includes around 319 species and 35 genera distributed in all biogeographical realms (Oswald and Machado 2018). Nine genera are recognized in the New World (Hoffman 1992, 2002) of which *Climaciella* Enderlein, 1910 contains nine species distributed from Southern Canada to northern Argentina including Cuba, Puerto Rico, and Dominican Republic (Hoffman 2002; Hoffman et al. 2017; Ardila-Camacho et al. 2018), plus an extinct species from France, badly preserved as a compression fossil from the late Oligocene (Nel 1988). The generic assignation of the fossil species was questioned by Hoffman (1992) based on venational characters, who proposed that it should be included into its own genus. The morphological characters supporting *Climaciella* as a monophylum include antennal flagellomeres along middle portion of flagellum ≥ 3× as wide as long in anterior view, lateral parapsidal suture (mesoscutal furrow *sensu*Hoffman (1992)) obsolete, and the female gonocoxites and gonapophyses VIII (both referred as sternite VIII by Hoffman (1992)) separated by thin membranous strip. Nevertheless, according to Hoffman (1992), these characters are also present in genera from other biogeographic regions such as *Euclimacia* Enderlein, 1910, *Pseudoclimaciella* Handschin, 1960, *Asperala* Lambkin, 1986, *Mantispilla* Enderlein, 1910, and *Spaminta* Lambkin, 1986, and the monophyly of this genus would be probably supported by characters of the first instar larva.

The biology of *Climaciella* is known mostly from studies performed with *C.brunnea* (Say, 1824), a widely distributed species composed of a complex of morphs (or subspecies), which mimic polistine wasps and occur through North America, Mexico, and Central America (Opler 1981; Batra 1972; Redborg and Macleod 1983; LaSalle 1986). According to Opler (1981), the Batesian mimicry of this species probably evolved because mimics and polistine models have similar habitats and habits. Adults of this species are diurnal and are often found on flowers or foliage, ambushing insect prey. Hoffman (1936) described some attributes of the behavior and morphology of the females, egg, and first instar larva, while Hoffman and Brushwein (1992) described the morphology of the first instar larva. Batra (1972) described the courtship, mating, and ovipositing behavior as well as the behavior of the first instar larvae. Additionally, this species was reported feeding on plant exudates, and *Polistesfuscatusutahensis* (Hayward, 1933) was determined as the model for the population studied by the author. In Costa Rica, the proportion of five different morphs of *C.brunnea* mimicking different species of Polistinae: *Polistesinstabilis* Saussure, 1853, *P.canadensis* (Linnaeus), 1758, *P.carnifex* (Fabricius), 1775, *P.erytrocephalus* (Latreille), 1813, and *Synoecaseptentrionalis* (Richards), 1978, varied among three different localities and could be related to differential abundance and aggressiveness of the models at each site (Opler 1981). Defensive behavior and displaying of the wasp-like warning colors, plus its capacity as occasional pollinators, was described by Boyden (1983).

After hatching, the first instar larva of *C.brunnea* adopts a vertical posture with the aid of the suctorial eversible process at the end of the abdomen while it maintains its legs extended (Batra 1972; Redborg and MacLeod 1983). Such questing behavior allows the larvae to board potential hosts that pass close by (Redborg and MacLeod 1983). This means that this species exhibits a phoretic behavior, in which the larvae first feed on spider hemolymph near the membranous areas of the carapace of the spider, and once the spider produces an egg-sac, they must reach the eggs before they are entirely encased with silk (Redborg and MacLeod 1983). This sequence is necessary for the larvae to be able to start feeding on the eggs, so it is classified as an obligate spider border, unable to penetrate egg-sacs like other genera of Mantispinae (Redborg and MacLeod 1983). As the main objective of the larvae is to enter an egg-sac during its construction, larvae of this species are able to move from male to female spiders during copulation (Scheffer 1992). *Climaciellabrunnea* has been found in the nature associated with species of the family Lycosidae, although laboratory experiments have shown that the larvae can board other spider families (e.g., Agelenidae and Salticidae), and complete their development (Redborg and MacLeod 1983; LaSalle 1986; Redborg and Redborg 2020; Snyman et al. 2020). Nonetheless, two morphs of *Climaciella* were found attacking egg sacs of Ctenidae and Araneidae in Panama (Miranda 2007), one of them probably by *Climaciellaporosa* Hoffman, 2002 (Ardila-Camacho and García 2015).

The taxonomy of the genus *Climaciella* was addressed in the classical studies of Mantispidae by Handschin (1960), and the works by Penny (1982) and Penny and da Costa (1983) which focused on the Amazonian fauna. Recent studies by Hoffman (2002) and Hoffman et al. (2017) treated this genus in Costa Rica and the West Indies, respectively, while Ardila-Camacho and García (2015) and Ardila-Camacho et al. (2018) dealt with taxonomy of the group in Colombia. In French Guiana, Thouvenot (2010) published a key to genera of Mantispidae, and thus far, only *C.semihyalina* has been reported in this territory (Ohl 2004). Herein, we update the knowledge on the diversity of *Climaciella* in French Guiana, describing two new species, providing new records and present a key to species. Furthermore, a new species from the Colombian Andes previously recorded as *Climaciellaamapaensis* Penny, 1982 by Ardila-Camacho and García (2015) is herein proposed partly based on comparison with a specimen of this species from French Guiana.

## ﻿Materials and methods

**Figure 1.**
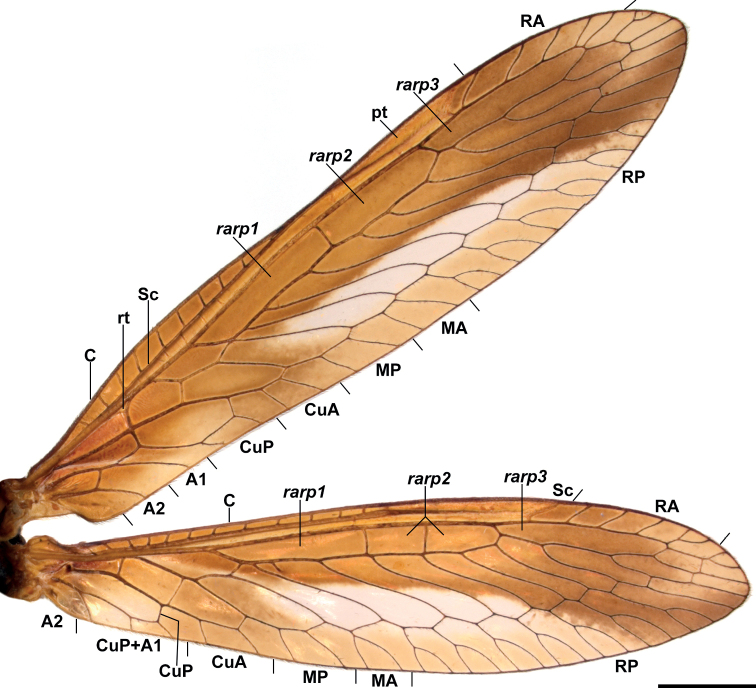
Wing venation in the genus *Climaciella*. *Climaciellabrunnea* (Say, 1824) (specimen from Jalisco, Mexico) as an example for the genus. Scale bar: 2 mm.

Specimens studied herein were collected in different localities of French Guiana between 2014 and 2016 using light traps, and then deposited in California State Collection of Arthropods, Sacramento CA (**CSCA**), Muséum national d’Histoire naturelle, Paris (**MNHN**), and Colección Nacional de Insectos, Instituto de Biología UNAM, Mexico City (**CNIN**). An additional specimen from Colombia is deposited in the private collection Colección Efraín Henao (CEH-085), Villamaría, Colombia. Other collections referred in this contribution are: Coleção Entomológica Pe. Jesus Santiago Moure, Curitiba, Paraná (**DZUP**), Museum of Comparative Zoology, Harvard University, Cambridge (**MCZ**), Naturhistorisches Museum, Bern (**NMBS**), and Museum für Naturkunde der Humboldt-Universität, Berlin (**ZMB**). Genitalia preparations were made by clearing the last abdominal segments in a hot solution of 10% potassium hydroxide (KOH). Residual alkaline solution was washed with distilled water and 80% ethyl alcohol. Then, the genitalia were stored in microvials filled with glycerin and mounted beneath the specimen. Wing venation was studied by spreading the wings of all specimens. Given the morphological peculiarities on the wing venation of *Climaciella*, the tracheation on the major traces of the specimens was compared to that of *C.brunnea* (Fig. 1), as this latter species is often used as example of the genus. External morphology and cleared genitalia were examined using a stereomicroscope. High-resolution images were produced using an AxioCam MRc5 digital camera attached to a Zeiss AxioZoom V16 stereomicroscope. Series of photographs were stacked and processed with the software ZENpro201. Morphological terminology and homology followed Ardila-Camacho et al. (2021).

## ﻿Taxonomy

### ﻿Family Mantispidae Leach, 1815

**Subfamily Mantispinae Leach, 1815**

#### ﻿Genus Climaciella

Taxon classificationAnimaliaNeuropteraMantispidae

Enderlein, 1910

28928553-E8E1-51C7-B4EE-51B7CBACBF6C

##### Diagnosis.

*Climaciella* is distinguished from other mantispines of the New World by the antennal flagellomeres ≥ 3 times as wide as long in frontal view, the lateral parapsidal furrow not developed, and the female gonocoxites and gonapophyses VIII separated by a membranous strip. Additionally, the membrane of the anterior half of the wings is amber colored, and all the species exhibit Batesian mimicry with polistine wasps.

##### Included species.

*Climaciellaamapaensis* Penny, 1982, *C.brunnea* (Say, 1824), *C.cubana* Enderlein, 1910, *C.elektroptera* sp. nov., *C.henrotayi* Nel, 1989, *C.nigriflava* sp. nov., *C.obtusa* Hoffman, 2002, *C.personata* (Stitz, 1913), *C.porosa* Hoffman, 2002, *C.rafaeli* Calle et al. in Ardila et al. 2018, *C.risaraldensis* sp. nov., *C.semihyalina* (Le Peletier de Saint Fargeau & Audinet-Serville in Latreille et al. 1825), and *C.tincta* (Navás, 1914).

### ﻿Key to the species of *Climaciella* Enderlein, 1910 from French Guiana

**Table:** 

1	Prothorax straight or at least only slightly bent medially in lateral view	**2**
–	Prothorax bent medially in lateral view	**3**
2	Body entirely black	***Climaciella* sp.**
–	Body yellow with black stripes	***Climaciellaamapaensis* Penny, 1982**
3	Head and prothorax black; anterior half of the wings smoky or dark amber	**4**
–	Head and prothorax mostly yellow or entirely dark brown; wings with anterior half pale amber	**5**
4	Wings with anterior half smoky, the posterodistal margin of the pigmented area darker; abdomen orange	***Climaciellatincta* (Navás, 1914)**
–	Wings with anterior half dark amber and hyaline posterodistal, triangular area; abdomen black	***Climaciellasemihyalina* (Le Peletier de Saint-Fargeau & Audinet-Serville, 1825)**
5	Body mostly yellow with dark brown stripes and marks	***Climaciellanigriflava* sp. nov.**
–	Body entirely dark brown	***Climaciellaelektroptera* sp. nov.**

#### ﻿ Climaciella

Taxon classificationAnimaliaNeuropteraMantispidae

sp.

0B69D258-F367-5187-B870-DF02BF5E6579

Fig. 2A

##### Specimens examined.

**French Guiana: Roura**, Montagne des Chevaux, Carrière du Galion, Créte avec foret sur quartzite érodée, 04°44'31.54"N, 52°25'53.02"W, 16.V.2015, automatic light trap (blue) (1♀ CNIN).

##### Remarks.

**Figure 2.**
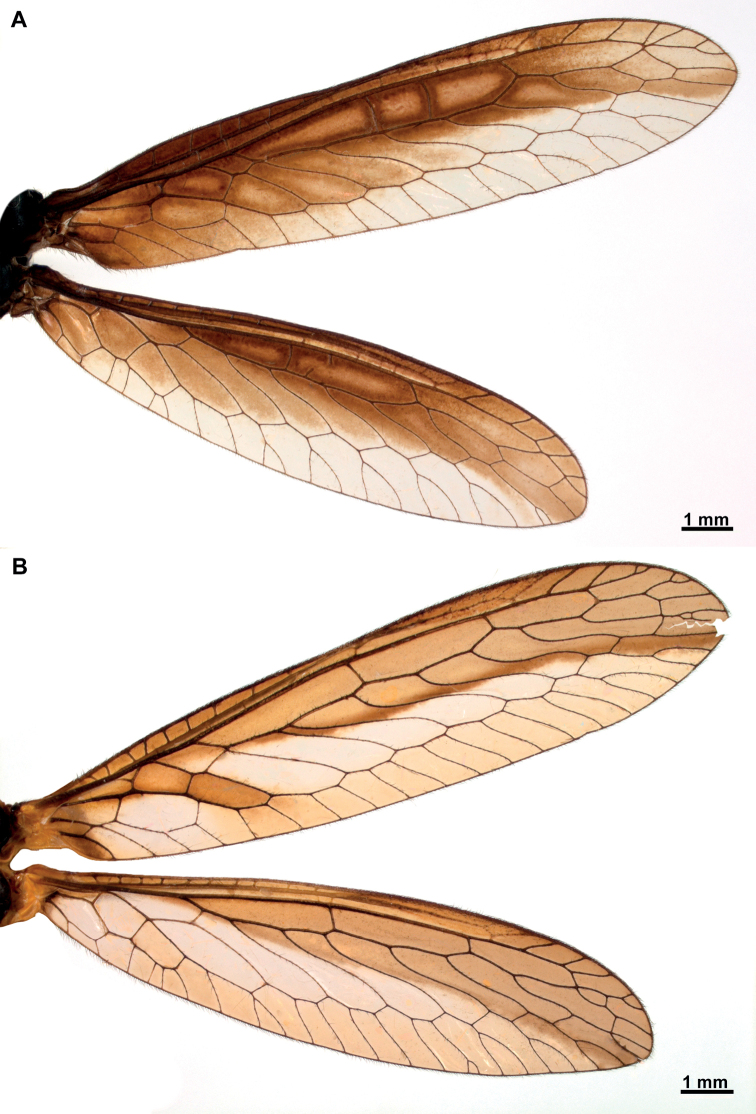
Wings of *Climaciella* species from French Guiana **A***Climaciella* sp. **B***Climaciellaamapaensis* Penny, 1982.

Despite this species exhibits a similar coloration pattern as *C.semihyalina*, the straighter prothorax and the wing pattern easily differentiate the unknown species. Compared to *C.semihyalina*, the anterior half of both wings of the specimen examined have paler areas inside the cells and wing apexes (Fig. 2A). In *C.semihyalina*, these same areas are noticeably darker and uniformly colored. Furthermore, the infuscation on the posterior half of both wings differ in their overall extension, shape and intensity between both species. The cited specimen examined herein probably represents a new species; however, as a single female only was available for this study, we opted to not describe this species until male specimens are available to confirm this hypothesis.

##### Distribution.

French Guiana.

#### ﻿ Climaciella amapaensis

Taxon classificationAnimaliaNeuropteraMantispidae

Penny, 1982

8AA0D132-F7AB-5B8D-A2B1-64C6E94C074C

Fig. 2B

Climaciella amapaensis Penny, 1982: 450. Holotype: male, Brazil (DZUP).

##### Specimens examined.

**French Guiana: Maripasoula**, Mitaraka, Contreforts du Mitaraka, crique Alama, Foret vallonnée au pied d’inselbergs, 20.VIII.2015, automatic light trap (GemLight) (1 sex? CNIN).

##### Diagnosis.

Records of *Climaciellaamapaensis* are scarce, but this species can be easily recognized by its coloration pattern that consists of a yellow body with black stripes and marks. Even though a similar color pattern is expressed by *C.nigriflava* sp. nov., *C.cubana*, *C.risaraldensis* sp. nov., and some morphs of *C.brunnea*, this species is distinguished because on the head there are two transversal bands at the level of the antennal insertion and on the upper portion of the region of the vertex, the compound eyes are enlarged and the region of the gena is narrow. In addition, the prothorax is straight in lateral view with longitudinal, lateral dark brown bands. This species is also unique within the genus due to the presence of narrow forefemur with thin processes. On the male genitalia, the apex of the gonocoxites X is not forked and blade-shaped, and the hypomeres are present as a single granule-shaped sclerite on the gonostyli X membrane laterally on each side.

##### Remarks.

This species is herein reported from French Guiana for the first time. In the original description, this species was reported from Brazil (Amapá) (Penny 1982), and then Hoffman (1992) provided a further record from Peru (Cusco). Recently, Ardila-Camacho and García (2015) provided a redescription and the first record from Colombia (Risaralda); however, this species actually represents a new species despite its noticeably similitude in coloration and morphology.

##### Distribution.

Brazil, French Guiana, Peru.

#### ﻿ Climaciella elektroptera

Taxon classificationAnimaliaNeuropteraMantispidae

Ardila-Camacho, Winterton & Contreras-Ramos sp. nov.

FD815F58-DF39-5ED3-BA7C-B4F42A847613

https://zoobank.org/8B20AC08-D64E-40FA-B4DD-1F1A4B48A5EC

Figs 3 , 4 , 5

##### Type material.

***Holotype*** ♂, **French Guiana: Maripasoula/Camopi**, Mont Itupé, Massif tabulaire, Pente oust (600 m), 28.XI.2014, light trap (MNHN). ***Paratypes*.** Same data as holotype (1♀ CNIN, 2♀ CSCA).

##### Diagnosis.

This species is easily differentiated from its congeners based on its uniformly dark brown body coloration. The prothorax is bent medially like in *C.semihyalina* and *C.obtusa* and lacks a prominent hump as in *C.porosa*. The wing coloration pattern is similar to that of *C.porosa*, with the anterior half of both wings pale amber. On the male genitalia, the gonocoxites X are bifid at the apex, and the gonostyli X membrane is set with a granule-shaped hypomere laterally at each side. Additionally, the gonostyli X are ribbon-shaped and the female spermatheca is relatively short and simple.

##### Description.

***Measurements*.** Head width: 2.9‒3.2 mm; Head length: 2.7‒3.4 mm; Prothorax length: 3.4‒3.9 mm; Forefemur length: 4.1‒6.4 mm; Forefemur maximum width: 1.7‒2.1 mm; Forewing length: 15.4‒16.5‒18.8 mm; Forewing maximum width: 3.5‒4.1 mm; Hindwing length: 13.3‒15.8 mm; Hindwing maximum width: 3.2‒3.9 mm.

**Figure 3.**
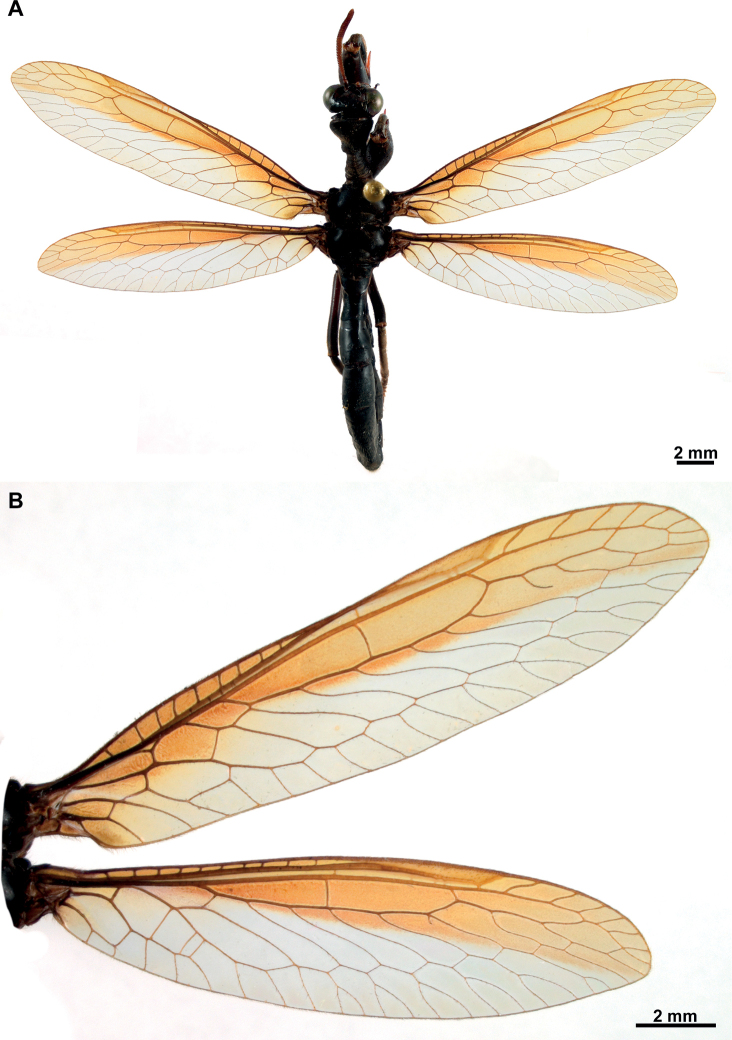
*Climaciellaelektroptera* sp. nov. **A** female habitus, dorsal **B** fore- and hindwing.

**Figure 4.**
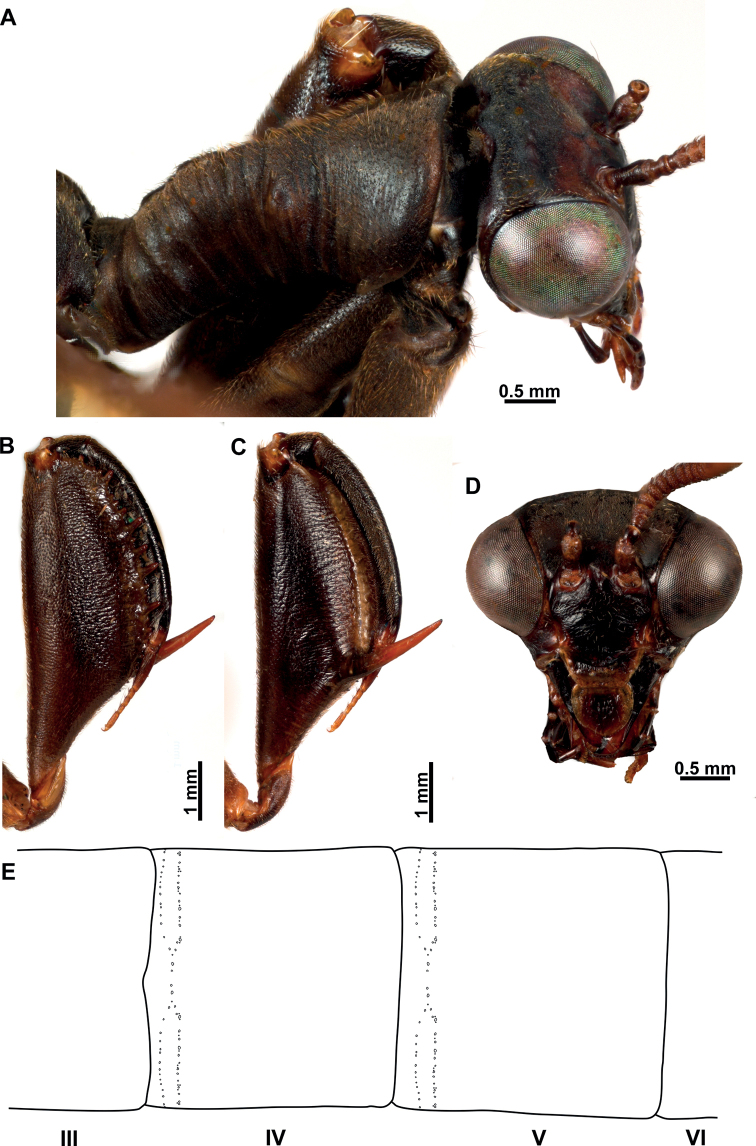
*Climaciellaelektroptera* sp. nov. **A** prothorax and head, dorsolateral view **B** foreleg, posterior surface **C** same, anterior surface **D** head, frontal view **E** male pregenital abdominal apparatus.

***Coloration*** (Figs 3, 4A–D). ***Head*.** Head capsule completely dark brown; antenna brown; flagellomeres with dark brown setae. Mouthparts dark brown. ***Thorax*.** Prothorax uniformly dark brown. ***Foreleg*.** Dark brown. ***Mid- and hind leg*.** Uniformly dark brown. ***Wings*.** Forewing membrane with anterior half and wing base pale brown, posterior half pale amber; venation brown, with paler and darker areas. Hind wing with costal, subcostal and radial fields, and *1r-m* and area adjacent to RP branches pale brown; remaining membrane pale amber; venation brown, with paler and darker areas. ***Abdomen*.** Dark brown, except pleural region of abdominal base cream.

***Morphology*** (Figs 3, 4). ***Head*.** Vertex region convex above compound eyes; compound eyes as wide as 0.75 of interocular distance at antennal insertion level. Antennal scape 2× as long as wide; pedicel as long as wide; basal flagellomere 1.5× as long as wide, the rest discoidal, with 31‒33 flagellomeres, all covered with fine, short setae. Frons quadrangular, frontoclypeal ridge barely perceptible; clypeus trapezoidal, labrum ovoid; mandible elongate; region of the gena as long as clypeus; maxillary palpus with basal palpomere slightly longer than wide; second palpomere 2.5× as long as wide; third and fourth palpomere 3× as long as wide; fifth palpomere as long as third. Submentum rectangular; labial palpus with first palpomere 3× as long as wide, second palpomere 5× as long as wide, third palpomere slightly longer than second; palpimacula groove-shaped. ***Thorax*.** Pronotum tubular, 4× as long as wide at maculae, slightly bent at mid-length in lateral view; medial region coarsely wrinkled; dorsal surface densely covered with fine, short setae; postfurcasternum narrowly ovoid. Mesonotum as long as wide, anterolaterally produced; lateral parapsidal suture obsolete; entire surface covered with fine, short setae; metanotum rectangular, scutum glabrous, scutellum with fine, short setae. Pteropleuron with fine, short setae. ***Foreleg*.** Coxa cylindrical, elongate, with proximal ring-shaped sulcus; anterior and posterior surface with abundant fine, short setae distal to proximal sulcus; trochanter ovoid; femur semi-triangular, robust, with abundant fine, short setae; posteroventral row of processes present on distal 2/3 of femur length; medial region of the row with two primary processes, the rest of the row with alternate secondary, tertiary, and quaternary processes, and numerous Stitz organs arising from reduced processes. Anteroventral row of processes reduced to a prominent major process. Tibia short and arched, reaching the major process of femur; anterior surface covered with abundant, clavate setae; distal margin acutely produced; closing surface with abundant trichoid setae. Basitarsus subconical, as long as eutarsus, with abundant trichoid setae on closing surface; pretarsus reduced to a single, simple claw. ***Mid- and hind leg*.** Coxae and trochanter subconical; femora tubular, that of hind leg slightly longer than on mid leg; tibiae elongate, thin, that of hind leg longer than on mid leg; tibial spurs well-developed. Tarsi with basitarsus slightly longer than eutarsus; pretarsal claws with six apical denticles. ***Wings*.** Forewing elongate and narrow; costal space narrow with seven or eight subcostal veinlets; Sc vein running close to C on area adjacent to pterostigma, and not approaching or touching RA at pterostigma level; pterostigma wedge-shaped; subcostal space with five or six crossveins on medial region, five or six crossveins on substigmal area. Radial space with two crossveins; one or two RP branches arising from *rarp1*, three or four from *rarp2*, 1‒3 from *rarp3*; six or seven gradate crossveins present. Media vein proximally fused to R, but inflexed forming a radial triangle; M diverging from R close R fork; M fork opposite to R fork; MA proximally fused to first RP stem for a short distance or connected to it through a short crossvein, forming a trapezoidal *rm2*. Cubitus forked near the level of radial triangle. Anal veins simple. Hind wing similar to forewing but shorter; costal space narrow, with nine subcostal veinlets; C and Sc not fused, running subparallel until pterostigma; pterostigma wedge-shaped; subcostal space with five crossveins on distal region. Radial space with three crossveins; two RP branches arising from *rarp1*, two or three from *rarp2*, one or two from *rarp3*; 5‒7 gradate crossveins present. Media vein basally fused to R, forked slightly before level of R fork. Cubitus forked slightly beyond the level of 1m-cu, CuA terminating on posterior wing margin at level of 1r-m, CuP distally fused to anterior branch of A1. ***Abdomen*.** Male tergites IV and V with two parallel rows of pores anterolaterally on each side which converge on inner end, with 28‒33 pores on each couple of rows (Fig. 4E); intertergal membrane between segments IV‒VI expanded, apparently forming an eversible sac. In both sexes tergite III with two anteromedial glabrous marks; tergite IV with four glabrous marks, two medial and two lateral, all located at mid-length; tergites V and VI with a single glabrous mark, laterally on each side.

**Figure 5.**
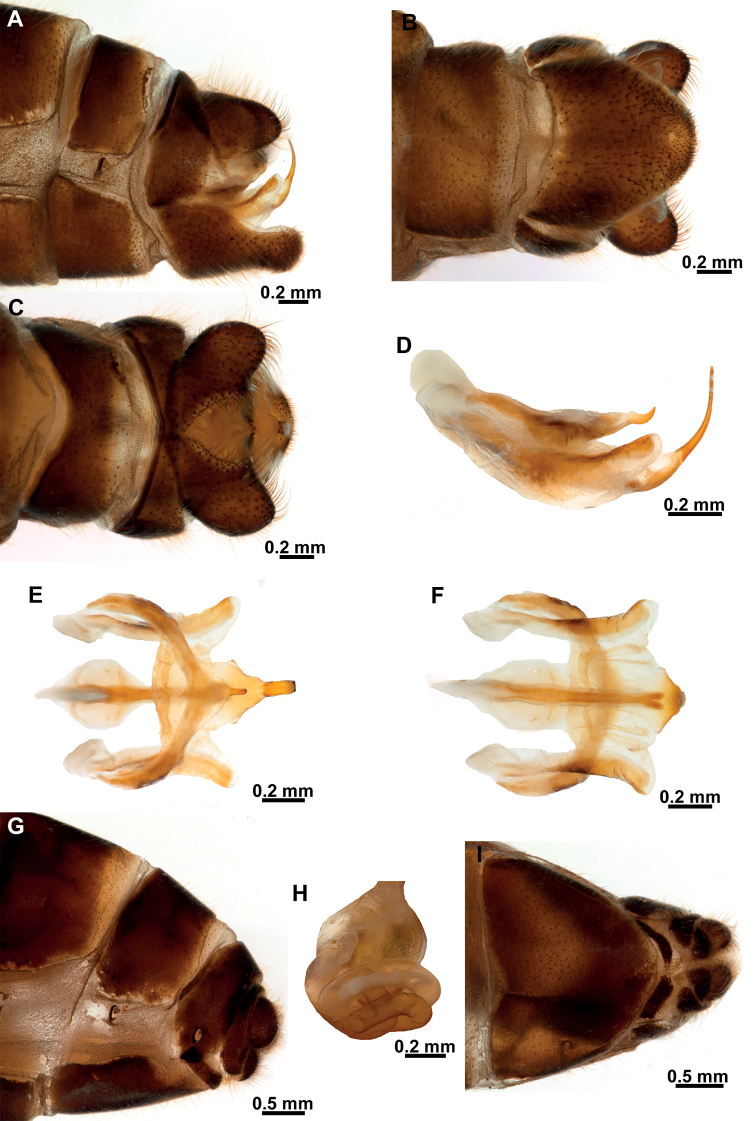
*Climaciellaelektroptera* sp. nov. **A** male terminalia, lateral **B** same, ventral **C** same, dorsal **D** male genitalia, lateral **E** same, dorsal **F** same, ventral **G** female terminalia, lateral **H** spermatheca **I** female terminalia, ventral.

***Male terminalia*** (Fig. 5A–F). Tergite IX half-ring shaped, medially narrower than laterally; sternite IX setose, approximately pentagonal, posteromedial region slightly protuberant. Gonocoxite IX elongated, curved, blunt on both apexes, anterior apex somewhat expanded, posterior apex curved outwards. Ectoproct ovoid, ventromedial lobe with around 42 stout setae. Gonocoxites X forming an elongate, gently curved sclerite, which is slightly expanded towards anterior apex, posterior apex bifid; gonostyli X membrane ventrally with triangular slightly sclerotized area, covered with microspinulae and with lateral granule-shaped hypomere; gonostyli X elongated, ribbon-shaped; entire surface with microspinulae. Gonocoxites XI arch-shaped, median lobe, short, hook-shaped.

***Female terminalia*** (Fig. 5G–I). Sternite VII (or gonocoxites VII) enlarged, pentagonal. Tergite VIII half-ring shaped, approximately as wide medially as laterally, enclosing the spiracle of the segment; gonocoxites VIII narrow, bar-shaped, subparallel-sided, slightly narrower at middle; gonapophyses VIII forming a bilobed, setose sclerite, connected to posteromedial region of gonocoxites VIII. Tergite IX half-ring shaped, posterolaterally connected to gonocoxites IX; gonocoxite IX a small and ovoid sclerite; gonapophyses IX probably represented by small, sclerotized areas on the anteroventral area of gonocoxites IX. Ectoprocts paired, ovoid. Bursa copulatrix short; spermatheca simple, entangled, with the same thickness on the different sections; fertilization canal duct and fertilization canal short.

##### Etymology.

The specific name of this species is from the Greek *ἤλεκτρον* (*ḗlektron*) meaning amber, and *πτερόν* (*pteron*), meaning wing, in allusion to the coloration of the wings of this species.

##### Remarks.

This new species is similar to *C.porosa* based on its general coloration; however, the bent prothorax and the lack of a hump on the medial region of the pronotum quickly differentiate it. Another species similar to *C.elektroptera* sp. nov. is *C.personata* from Bolivia, although the latter has a more patterned body coloration, and the posterior limit of the amber anterior area of the wings is darker and noticeably marked. Based on the bent prothorax, *C.elektroptera* sp. nov. is probably closely related to *C.obtusa*, *C.semihyalina*, *C.personata*, *C.rafaeli*, and *C.nigriflava* sp. nov.

Based on the coloration pattern, size, and distribution of *C.elektroptera* sp. nov., the possible wasp models for this species could be *Agelaiaangulata* (Fabricius, 1804) or *Polistesdeceptor* Schulz, 1905 (R. Lopes, pers. comm. 2022)

##### Distribution.

French Guiana.

#### ﻿ Climaciella nigriflava

Taxon classificationAnimaliaNeuropteraMantispidae

Ardila-Camacho, Winterton & Contreras-Ramos sp. nov.

A52BA953-8F5C-5BEA-BAB0-7CC44E80C032

https://zoobank.org/01623019-404A-4378-BFE1-7363E84A18CE

Figs 6 , 7 , 8

##### Type material.

***Holotype*** ♂, **French Guiana: Roura**, Montagne des Chevaux, Carrière du Galion, Crete avec foret sur quartzite érodée, 04°44'31.54"N, 52°25'53.02"W, 22.II.2016, automatic light trap (blue) (MNHN). ***Paratypes*. French Guiana: Maripasoula**, Mitaraka, Contreforts du Mitaraka, crique Alama, Foret vallonnée au pied d’inselbergs, 18.III.2015, light trap (1♀ CNIN); same data but 20.VIII.2015, automatic light trap (blue) (1♂ CSCA). **Roura**, Montagne des Chevaux, Carrière du Galion, Crête avec forêt sur quartzite érodée, 4°44'31,54"N, 52°25'53,02"W, 25.IV.2015, automatic light trap (blue) (1♂ CSCA).

##### Diagnosis.

This new species has a similar body coloration pattern as *C.amapaensis*, *C.cubana*, and certain morphs of *C.brunnea*. However, the bent prothorax at midlength in lateral view distinguishes the new species from the aforementioned ones. Furthermore, the head in *C.nigriflava* sp. nov. is basically entirely yellow, while the remaining species with similar coloration exhibit dark stripes or marks. The coloration of the femur is also similar to that of *C.amapaensis*, yet the more robust femur with thickened processes distinguishes *C.nigriflava* sp. nov. from the former. Moreover, the wing coloration of this species is similar to that of *C.porosa* and *C.elektroptera* sp. nov. In the male genitalia, the gonocoxites X have the posterior apex truncate, the hypomeres are absent, and the gonostyli X are elongated, ribbon-shaped with the apex forming an obtuse angle.

##### Description.

***Measurements*.** Head width: 3.0‒3.1 mm; Head length: 2.6‒3.0 mm; Prothorax length: 3.6‒3.8 mm; Forefemur length: 5.2 mm; Forefemur maximum width: 1.9 mm; Forewing length: 15.9‒16.5 mm; Forewing maximum width: 3.5‒3.7 mm; Hindwing length: 13.5‒14.1 mm; Hindwing maximum width: 3.2‒3.5 mm.

**Figure 6.**
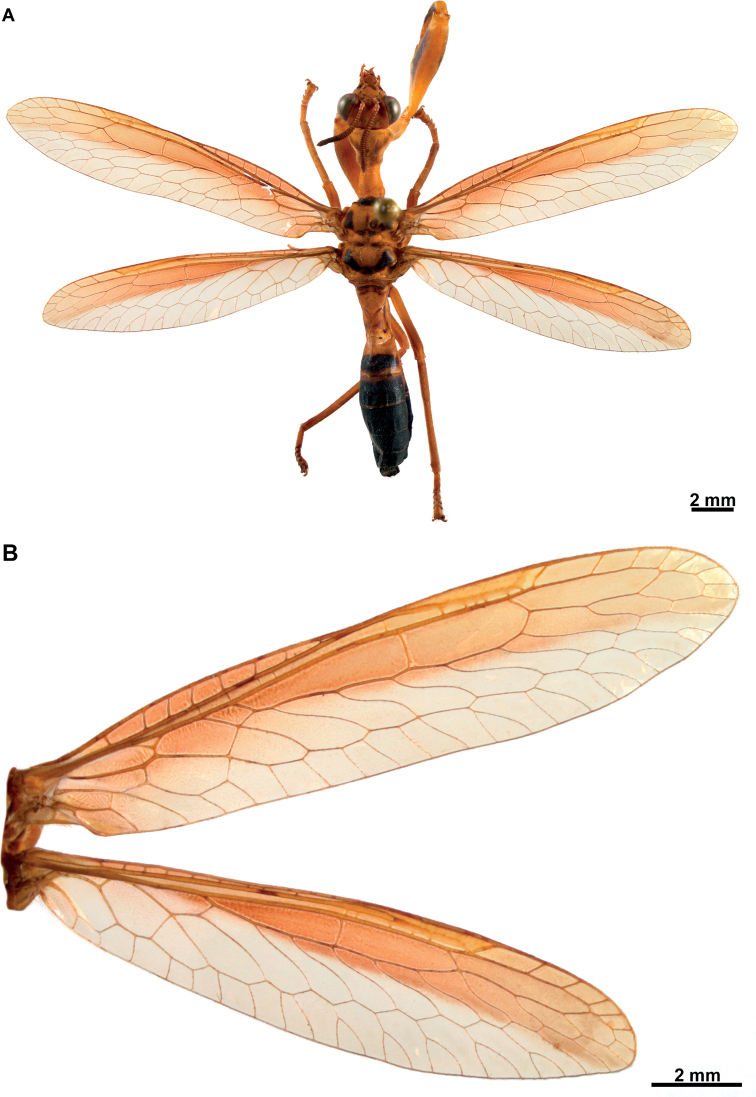
*Climaciellanigriflava* sp. nov. **A** female habitus, dorsal **B** fore- and hindwing.

**Figure 7.**
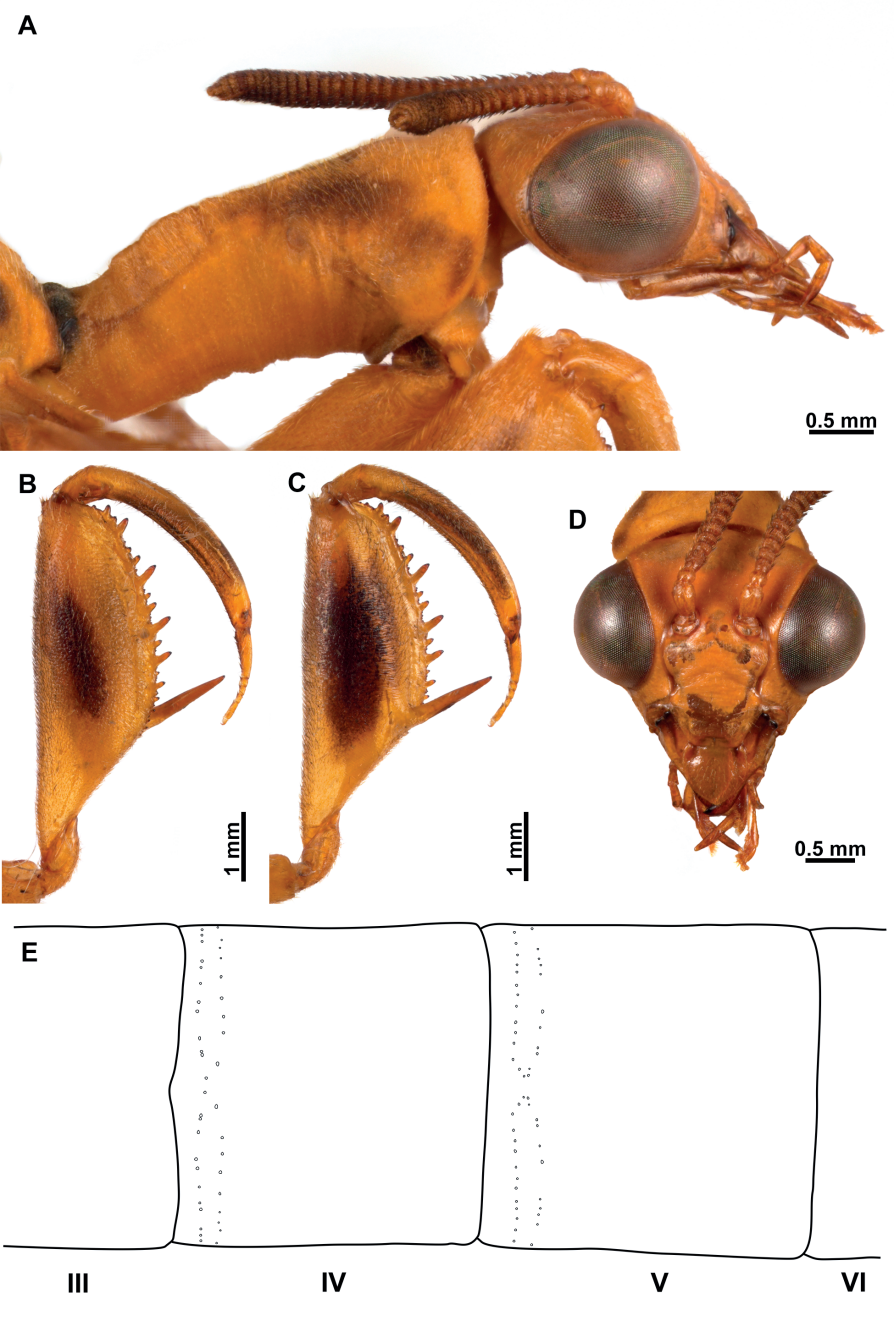
*Climaciellanigriflava* sp. nov. **A** prothorax and head, lateral view **B** foreleg, posterior surface **C** same, anterior surface **D** head, frontal view **E** male pregenital abdominal apparatus.

***Coloration*** (Figs 6, 7A–D). ***Head.*** Head capsule completely yellow; antenna yellow, sometimes pale brown towards the flagellar apex; flagellomeres with dark brown setae. Clypeus and labrum yellow; mandible pale brown; maxilla and labium pale brown. ***Thorax*.** Pronotum yellow, with pale brown, lateral areas on the prozona extended from macula to anterior pronotal margin; postfurcasternum yellow. Mesoscutum yellow with brown anterior margin and lateral spots; mesoscutellum yellow with brown, medial area. Metascutum yellow with lateral brown marks; mesoscutellum with anterior half brown, posterior half yellow. Pteropleuron yellow with brown on area adjacent to anapleural cleft and paracoxal suture. ***Foreleg*.** Coxa and trochanter yellow; femur yellow with small brown mark on medial region of posterior surface, and larger brown area on anterior surface; closing surface with processes yellow but changing to amber towards the apex; tibia mostly yellow with pale brown areas, except anterior surface with darker area. Tarsus pale brown. ***Mid- and hind leg*.** Coxa of both legs yellow to pale brown; trochanter, femur, tibia, and tarsus pale brown. ***Wings*.** Forewing membrane with anterior half and wing base pale brown, posterior half pale amber; venation brown, with paler and darker areas. Hind wing with costal, subcostal and radial fields, and *1r-m* and area adjacent to RP branches pale brown; remaining membrane pale amber; venation brown, with paler and darker areas. ***Abdomen*.** Abdominal segments I and II yellow with pale brown areas; male tergites III‒V with striped pattern, exhibiting yellow anterior band and dark brown band on each segment; female tergites III yellow with broad, transversal dark brown band, tergite IV mostly dark brown; remaining tergites on both sexes completely dark brown. Male sternite III yellow, sternites IV and V brown, the remainder dark brown; female sternite III yellow, sternite IV with anterior yellow band and posterior brown band, remaining sternites dark brown.

***Morphology*** (Figs 6, 7). ***Head*.** Vertex region convex above compound eyes; compound eyes as wide as 0.75 of interocular distance at antennal insertion level. Antennal scape 1.5× as long as wide; pedicel as long as wide; basal flagellomere as long as wide, the rest discoidal, with 32 or 33 flagellomeres, all covered with fine, short setae. Frons subquadrate, frontoclypeal ridge barely perceptible; clypeus trapezoidal, labrum ovoid; mandible elongate; region of the gena as long as clypeus; maxillary palpus with basal palpomere slightly wider than long; second palpomere 2× as long as wide; third and fourth palpomere subequal, both 2.5× as long as wide; fifth palpomere 1.2× as long as fourth. Submentum rectangular; labial palpus with first palpomere 4× as long as wide, second palpomere 5× as long as wide, third palpomere slightly longer than second; palpimacula groove-shaped. ***Thorax*.** Pronotum tubular, 3.5× as long as wide at maculae, slightly bent at mid-length in lateral view; medial region coarsely wrinkled; dorsal surface densely covered with fine, short setae; postfurcasternum narrowly ovoid. Mesonotum as long as wide, anterolaterally produced; lateral parapsidal suture obsolete; entire surface covered with fine, short setae; metanotum rectangular, scutum glabrous, scutellum with fine, short setae. Pteropleuron with fine, short setae. ***Foreleg*.** Coxa cylindrical, elongate, with proximal ring-shaped sulcus; anterior and posterior surface with abundant fine setae distal to proximal sulcus; trochanter ovoid; femur semi-triangular, robust, with abundant fine, short setae; posteroventral row of processes present on distal 2/3 of femur length; medial region of the row with two primary processes, the remainder of the row with alternate secondary, tertiary, and quaternary processes, and numerous Stitz organs arising from reduced processes. Anteroventral row of processes reduced to a prominent major process. Tibia short and arched, reaching the major process of femur; anterior surface covered with abundant, clavate setae; distal margin acutely produced; closing surface with abundant trichoid setae. Basitarsus subconical, as long as eutarsus, with abundant trichoid setae on closing surface; pretarsus reduced to a single, simple claw. ***Mid- and hind leg*.** Coxae and trochanter subconical; femora tubular, that of hind leg slightly longer than on mid leg; tibiae elongate, thin, that of hind leg longer than on mid leg; tibial spurs well-developed. Tarsi with basitarsus slightly shorter than eutarsus; pretarsal claws with five or six apical denticles. ***Wings*.** Forewing elongate and narrow; costal space narrow with 6‒8 subcostal veinlets; Sc vein running close to C on area adjacent to pterostigma, and not approaching or touching RA at pterostigmal level; pterostigma wedge-shaped; subcostal space with four or five crossveins on medial region, six crossveins on substigmal area. Radial space with three crossveins; one or two RP branches arising from *rarp1*, two or three from *rarp2*, and two or three from *rarp3*; 6‒8 gradate crossveins present. Media vein proximally fused to R, but inflexed forming a radial triangle; diverging from R close R fork; M fork opposite to R fork; MA proximally fused to first RP stem for a short distance, forming a trapezoidal *rm2*. Cubitus forked near the level of radial triangle. Anal veins simple. Hind wing similar to forewing but shorter; subcostal space narrow, with eight or nine subcostal veinlets; C and Sc not fused, running subparallel until pterostigma; pterostigma wedge-shaped; subcostal space with four or five crossveins on distal region. Radial space with three crossveins; two or three RP branches arising from *rarp1*, two or three from *rarp2*, 1‒3 from *rarp3*; 6‒8 gradate crossveins present. Media vein basally fused to R, forked slightly before level of R fork. Cubitus forked slightly beyond the level of 1m-cu, CuA terminating on posterior wing margin at level of 1r-m, CuP distally fused to anterior branch of A1. ***Abdomen*.** Male tergites IV and V with two parallel rows of pores anterolaterally on each side which converge on the inner end, with 22‒30 pores on each couple of rows (Fig. 7E); intertergal membrane between segments IV‒VI expanded, apparently forming an eversible sac. In both sexes tergite III with two anteromedial glabrous marks; tergite IV with four glabrous marks, two medial and two lateral, all located at mid-length; tergites V and VI with a single glabrous mark, laterally on each side.

**Figure 8.**
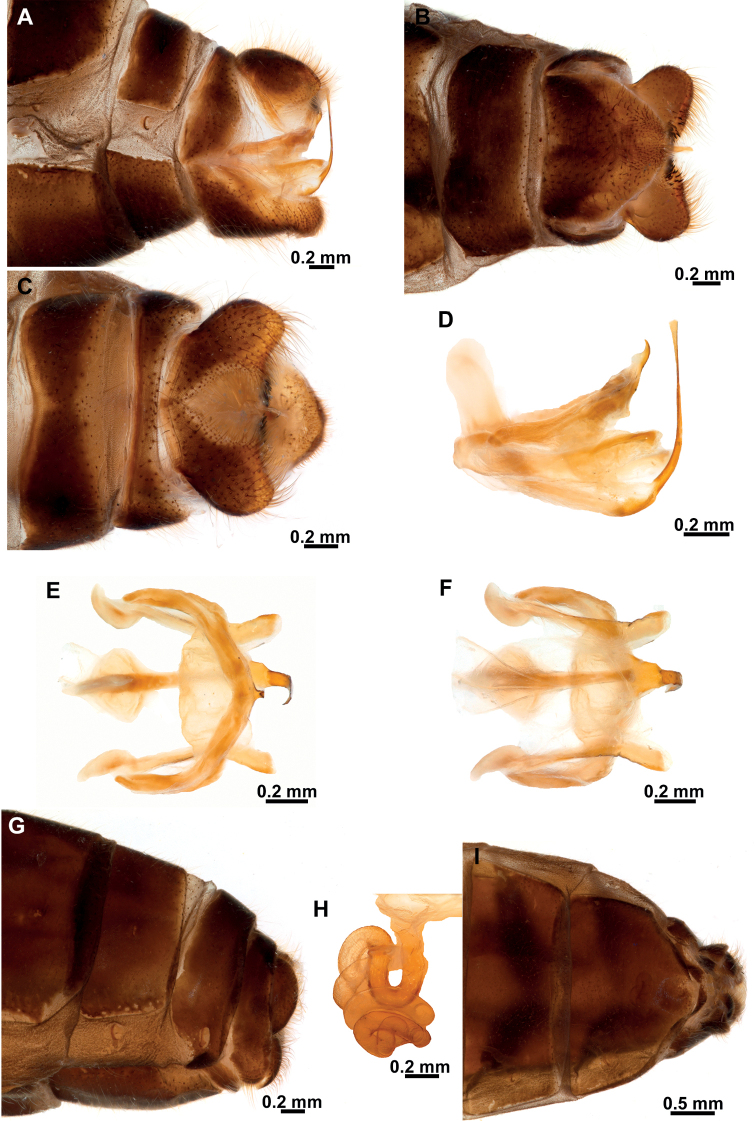
*Climaciellanigriflava* sp. nov. **A** male terminalia, lateral **B** same, ventral **C** same, dorsal **D** male genitalia, lateral **E** same, dorsal **F** same, ventral **G** female terminalia, lateral **H** spermatheca **I** female terminalia, ventral.

***Male terminalia*** (Fig. 8A–F). Tergite IX half-ring shaped, medially narrower than laterally; Sternite IX setose, approximately pentagonal, posteromedial region slightly protuberant. Gonocoxite IX elongated, sigmoid, blunt on both apexes, anterior apex somewhat expanded. Ectoproct ovoid, ventromedial lobe with around 36 stout setae. Gonocoxites X forming an elongate and arched sclerite, which is slightly expanded towards anterior apex, posterior apex truncate; gonostyli X membrane ventrally slightly sclerotized covered with microspinulae; gonostyli X elongated, ribbon-shaped, slightly shorter than gonocoxites X, apex forming an obtuse angle; entire surface with microspinulae. Gonocoxites XI arch-shaped, median lobe, short, hook-shaped.

***Female terminalia*** (Fig. 8G–I). Sternite VII (or gonocoxites VII) enlarged, U-shaped. Tergite VIII half-ring shaped, approximately as wide medially as laterally, enclosing the spiracle of the segment; gonocoxites VIII narrow, bar-shaped, subparallel-sided, slightly narrower at middle; gonapophyses VIII forming a rectangular, setose sclerite, connected to posteromedial region of gonocoxites VIII. Tergite IX half-ring shaped, posterolaterally connected to gonocoxites IX; gonocoxite IX a small and ovoid sclerite; gonapophyses IX probably represented by small, sclerotized areas on the anteroventral area of gonocoxites IX. Ectoprocts paired, ovoid. Bursa copulatrix short; spermatheca simple, spiral-shaped, progressively narrowed towards the apex; fertilization canal duct and fertilization canal short.

##### Etymology.

The specific epithet of this species is a combination of the Latin words *niger* which means black, and *flavus* meaning yellow, in allusion to the coloration pattern of the body of this species.

##### Remarks.

Based on the prothorax morphology, this new species appears to be closely related to *C.obtusa*, *C.semihyalina*, *C.personata*, *C.rafaeli*, and *C.elektroptera* sp. nov. The body coloration pattern of the new species with head and thorax predominantly yellow, and a large dark area on the abdomen indicates this species could be a mimic of the vespid wasps *Agelaiapallipes* (Olivier, 1792) or *Mischocyttaruscerberus* Ducke, 1918 in the case of smaller specimens, or *Polistestestaceicolor* Bequard, 1937 or *Agelaiatestacea* (Fabricius, 1804) in the case of larger specimens (R. Lopes, pers. comm. 2022).

#### ﻿ Climaciella semihyalina

Taxon classificationAnimaliaNeuropteraMantispidae

(Le Peletier de Saint-Fargeau & Audinet-Serville in Latreille et al. 1825)

AD1EF6E2-6D8E-52D5-8521-A8ED2289C585

Fig. 9A

Mantispa semihyalina Le Peletier de Saint-Fargeau & Audinet-Serville in Latreille et al. 1825: 270, sex not indicated. Holotype (or syntypes): sex unknown, Brazil (MNHN).
Mantispa chalybea Erichson, 1839: 160, sex not indicated. Syntypes: sex unknown, Brazil, Suriname (ZMB, MCZ). Synonymized by Enderlein 1910: 367.
Mantispa grandis ; Burmeister, 1839: 967. Not available with Burmeister as author. Synonymized by Westwood 1852: 253.
Nobrega tinctus Navás, 1914: 233, sex not indicated (see C.tinctus below). Holotype: sex unknown, Brazil (NHMUK). Synonymized by Penny 1982: 453. (non).

##### Specimens examined.

**French Guiana: Maripasoula**, Mitaraka, Contreforts du Mitaraka, crique Alama, Foret vallonnée au pied d’inselbergs, 12.III.2015, light trap (1♂ CNIN); same data but automatic light trap (blue) (1♂ CSCA).

##### Diagnosis.

The overall black coloration of the body of this species is shared with *C.obtusa*, *C.rafaeli*, and the *synoeca* morph of *C.brunnea*. However, the wing pattern of *C.semihyalina* easily separates it from the former two, whereas the bent prothorax distinguishes this species from the latter. This species is rapidly recognized by the dark amber anterior half of the wings, while in the posterior half, the basal region is pale amber, while a preapical triangular area is hyaline. On the male genitalia, the apex of the gonocoxites X is bifid, and the gonostyli X are spine-shaped.

**Figure 9.**
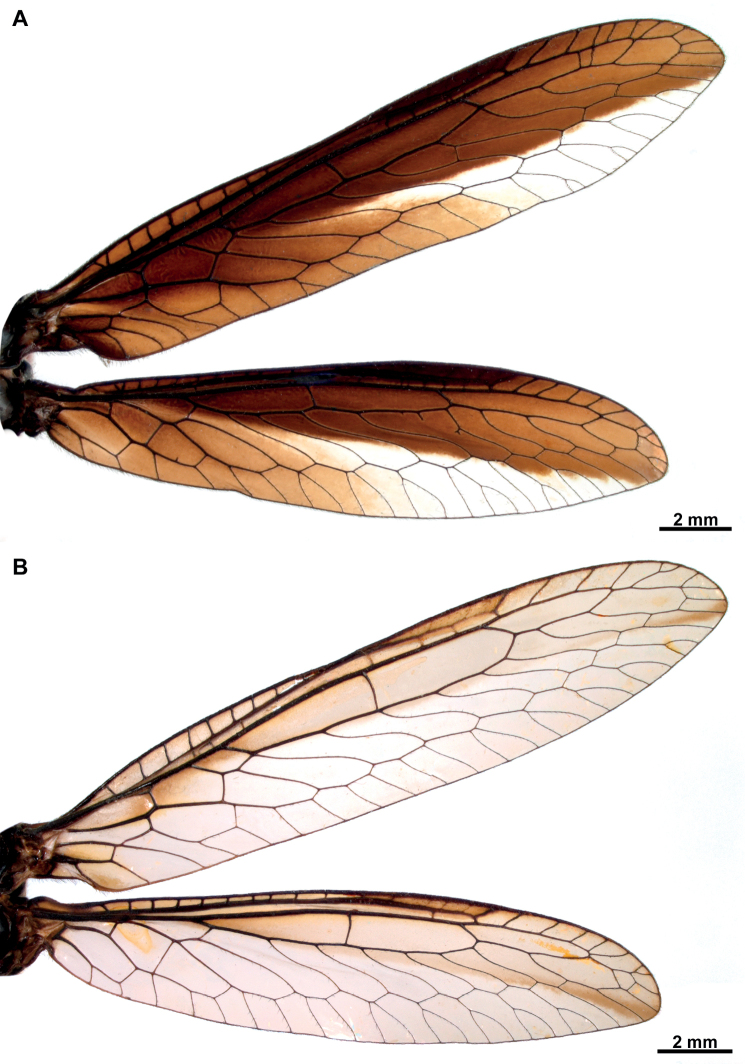
Wings of *Climaciella* species from French Guiana **A***Climaciellasemihyalina* (Le Peletier de Saint-Fargeau & Audinet-Serville in Latreille et al. 1825) **B***Climaciellatincta* (Navás, 1914).

##### Remarks.

*Climaciellasemihyalina*, is the most commonly encountered species of the genus in South America, being widely distributed in the Amazon. The bent prothorax together the robust forefemora of this species resemble the shape of the head of its potential models, i.e., the vespids *Polybiasimillima* Smith, 1862, *P.ignobilis* (Haliday, 1836) (Ardila-Camacho and García 2015), *Synoecasurinama* (Linnaeus, 1767), or *Polistesgoeldii* Ducke, 1904 (R. Lopes, pers. comm. 2022). The basal orange-reddish region of the forefemur apparently resembles the mandibles of the wasp model. This species was already known in French Guiana, with the first record from this territory published by Navás (1926). The wide distribution of this species from the Amazon to Argentina, calls into attention about the possibility that the different populations could represent actually different species.

##### Distribution.

Argentina, Bolivia, Brazil, Colombia, Ecuador, French Guiana, Paraguay, Peru, Suriname, Uruguay.

#### ﻿ Climaciella tincta

Taxon classificationAnimaliaNeuropteraMantispidae

(Navás, 1914)

B9519187-444E-587A-94C9-B8D526FCB987

Figs 9B , 10

Nobrega tinctus Navás, 1914: 233. Holotype: sex unknown, Brazil (NHMUK).
Climaciella duckei Navás, 1915: 196. Holotype: sex unknown, Peru (NMBS). Synonymized by Alvim, 2021: 31.
Climaciella tincta (Navás, 1914); Alvim 2021: 31.

##### Specimens examined.

**French Guiana: Maripassoula/Camopi**, Mont Itoupé, Massif tabulaire, Pente oust (600 m), 23.XI.2014, light trap (1♂, 1♀ CNIN); same data but 24.XI.2014, light trap (1♀ CSCA); same data but 29.XI.2014, light trap (2♀ CSCA). **Roura**, Montagne des Chevaux, Carrière du Galion, Crête avec forêt sur quartzite érodée, 4°44'31,54"N, 52°25'53,02"W, 10.X.2015, automatic light trap (blue) (1♀ CSCA).

##### Diagnosis.

This species has a distinctive coloration of the body with black head and thorax and orange abdomen. The prothorax is bent like in *C.semihyalina*, *C.obtusa*, *C.personata*, *C.rafaeli*, and the new species described herein. The wings have smoky anterior half, with the posterodistal margin of this pigmented area more marked. On the male genitalia, the apex of the gonocoxites X is bifid, the hypomeres are present as a lateral granule-shaped sclerite at each side, and the gonostyli X is elongated and narrow.

##### Remarks.

Based on the prothorax morphology, *C.tincta* is apparently closely related with *C.semihyalina*, *C.obtusa*, *C.personata*, *C.rafaeli*, *C.elektroptera* sp. nov., and *C.nigriflava* sp. nov. This species was originally described by Navás (1914) as *Nobregatinctus* Navás, 1914; however, Penny (1982) proposed this species as a synonym of *C.semihyalina* based on the coloration of the head and the thorax. It is noteworthy that the type of *Nobregatinctus* lacks an abdomen, so the coloration and genitalic morphology were unknown by Penny (1982). Furthermore, he considered the wing coloration of the type of *N.tinctus* as intraspecific variation. Hoffman (1992) revalidated this species and transferred it to the genus *Climaciella* after the examination of the type. Additionally, he synonymized *Climacielladuckei* Navás,1915 under *C.tincta*. These results were corroborated by Alvim (2021), who redescribed this species and provided illustrations of the male and female genitalia for the first time. Herein, we provide the first record of *C.tincta* from French Guiana.

**Figure 10.**
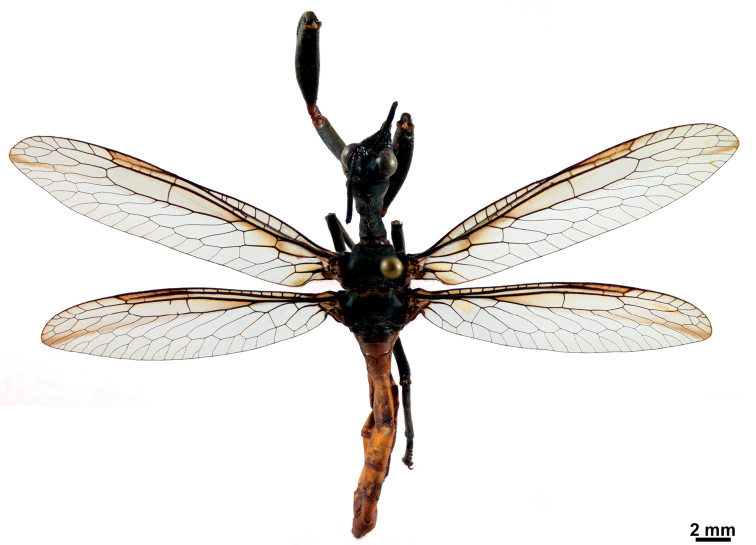
Habitus of female *Climaciellatincta* in dorsal view.

Based on the distribution and coloration pattern exhibited by *C.tincta* (Fig. 10), the possible models for this species could be *Polybiarejecta* (Fabricius, 1798) in the case of small specimens, while *Polybiadimidiata*, *Polistesoccipitalis* Ducke, 1904, and *Polistesbicolor* Fox, 1898 could be the models for larger specimens (R. Lopes, pers. comm. 2022).

##### Distribution.

Brazil, French Guiana.

#### ﻿ Climaciella risaraldensis

Taxon classificationAnimaliaNeuropteraMantispidae

Ardila-Camacho sp. nov.

9CCBA169-ABE0-5418-AAAA-E9AB48262440

https://zoobank.org/07366DDF-2CDC-416F-9D77-6DD21026F018

##### Type material.

***Holotype*** ♂, **Colombia**: **Risaralda**, Tatamá, Centro de visitantes planes de San Rafael, 5°4'20,87"N, 75°57'44,45"W, 2400 m, 10.VI.2010, F. Gaviria, entomological net (CEH-085).

##### Diagnosis.

This new species may be distinguished from the majority of the species in the genus by the general coloration pattern that is yellow with black stripes and marks. Furthermore, *C.risaraldensis* sp. nov. is easily separated from *C.nigriflava* sp. nov. by the black vertex region and the straight prothorax. On the other hand, this new species can be separated from *C.amapaensis* by having smaller compound eyes and broadened region of the gena, pronotum dark brown with small diffuse yellow areas, anterior half of the wing pale amber, the forked apex of the male gonocoxites X, and hypomeres present as two granules on each side of the gonostyli X membrane.

##### Description.

See Ardila-Camacho and García (2015).

##### Etymology.

This species is named after the department of Risaralda, a region of the central Andes of Colombia where the type locality of this species is located.

##### Remarks.

Based on the descriptions of Penny (1982) and Hoffman (1992), this species was initially identified as *C.amapaensis* by Ardila-Camacho and García (2015) as both have a quite similar coloration and patterning, and because the former authors did not provide enough details of the male pregenital, abdominal apparatus and genitalia. *Climaciellarisaraldensis* sp. nov. is noticeably morphologically differentiated from its Amazonian congeners exhibiting a yellow and black coloration pattern. Furthermore, this species is found at high elevation (2400 m) in the mountains of the Western Cordillera of Colombia, an area remarkably isolated from the Amazon.

Based on the morphology of the prothorax, this species is probably closely related to *C.amapaensis*, *C.brunnea*, *C.cubana*, and *C.porosa*. Among these, the new species is similar to certain morphs of *C.brunnea*; however the wing coloration, the male pregenital apparatus, and the genitalic morphology are markedly differentiated between both species.

##### Distribution.

Colombia.

## ﻿Discussion

In the present study, the number of species of *Climaciella* in French Guiana is increased from one to six, based on considerable collecting efforts performed in this small portion of the Amazon. The diversity of species in this territory is quite remarkable compared to other, larger Neotropical countries. In Colombia and Brazil for example, five species of this genus have been reported to date (Ardila-Camacho and García 2015; Ardila-Camacho et al. 2018; Alvim 2021). By contrast, in a smaller and well-sampled country such as Costa Rica, three species have been reported (Hoffman 2002). It is likely that the number of species recorded in the Neotropics is influenced by the collecting efforts, and a considerable diversity of this genus is expected to be found, primarily in countries of Northern South America, where this genus appears to have its higher diversity.

The two Guianese new species described herein are apparently closely related based on genitalic morphology. Both species appear to form a group of species together with *C.semihyalina*, *C.obtusa*, *C.rafaeli*, *C.personata*, and *C.tincta* based on the bent prothorax, a hypothesis supported in the cladistic analysis performed by Hoffman (1992). By contrast, *C.amapaensis*, *C.brunnea*, *C.cubana*, *C.porosa*, and *C.risaraldensis* sp. nov. form a group of species distinguished by a straight prothorax, although this still needs to be tested in a phylogenetic framework. In the phylogenetic analysis performed by Hoffman (1992), *C.cubana* was recovered as sister of the other species, while *C.brunnea* and *C.porosa* were recovered as sister based on the presence of five or more transverse rows of pores anterolaterally on tergite IV at each side, and the forked apex of the male gonocoxite IX.

*Climaciella* was recovered as sister of the remainder of mantispine genera of the New World by Hoffman (1992) based on morphological characters. This genus exhibits a series character states such as the MA of the FW fused or nearly fused (i.e., separated by short crossvein) to the RP stem and the overall morphology of the fusion between the CuP and the anterior branch of the A1 of the HW, which are shared with genera from other realms such as *Austroclimaciella* Handschin,1961, *Euclimacia*, *Pseudoclimaciella*, *Nampista* Navás, 1914, *Tuberonotha* Handschin, 1961, and *Mimetispa* Handschin, 1961 (Hoffman 1992; Snyman et al. 2012; Snyman et al. 2018). Interestingly, all these genera also express Batesian mimicry, begging the question if this attribute arose a single time in all these taxa or if it arose multiple times independently. Moreover, in the New World, the mimicry with vespid wasps is also expressed by *Entanoneura* and likely by *Paramantispa* Williner & Kormilev, 1958, of which the latter appears to be closely related to *Climaciella* based on venational characters, especially the close association between the MA and the RP stem of the FW. Furthermore, species of *Paramantispa* and *Climaciella* have relatively small compound eyes and broadened genal region, with the exception of *C.amapaensis* that has enlarged compound eyes and narrow genal region. Based on the work of Hoffman (1992), *Entanoneura* and *Paramantispa* are sister groups and are more related to other mantispine genera of the New World lacking Batesian mimicry. This would suggest, that at least, in the New World this condition arose independently in different genera.

## Supplementary Material

XML Treatment for Climaciella

XML Treatment for Climaciella

XML Treatment for Climaciella amapaensis

XML Treatment for Climaciella elektroptera

XML Treatment for Climaciella nigriflava

XML Treatment for Climaciella semihyalina

XML Treatment for Climaciella tincta

XML Treatment for Climaciella risaraldensis
